# Off the beaten path: A scoping review of how ‘rural’ is defined by the U.S. government for rural health promotion

**DOI:** 10.34172/hpp.2022.02

**Published:** 2022-05-29

**Authors:** Elisa M. Childs, Javier F. Boyas, Julianne R. Blackburn

**Affiliations:** ^1^School of Social Work, University of Georgia, 279 Williams St., Athens, GA, 30602, USA; ^2^School of Social Work and Human Services, Troy University, 112-D Wright Hall, Troy, AL, 36082, USA

**Keywords:** Rural dynamics, Rural health, Rural health services, Government regulation, Health promotion, Health equity, Health policy, Review, Classification

## Abstract

**Background:** Given the recognition that the U.S. government lacks a consensus definition of the word *rural*, the purpose of this scoping review was to uncover how the federal government defines the term and to establish a nuanced understanding of what criterion is used to designate an area as rural.

**Methods:** Arksey and O’Malley’s framework was used to synthesize, analyze, and summarize the existing literature. A multi-system search was conducted, and articles were screened for eligibility by two independent reviewers using pretested forms.

**Results:** Initially, 929 articles were screened that used the search terms *rural* and some variation of the word *definition*. After eliminating all ineligble studies, 49 documents were included in the final analysis. These documents revealed 33 federal definitions of *rural*. The majority of definitions centered on either population, population density, or urban integration provisions. Additionally, the analysis showed that the literature could be separated into two categories: how *rural* was defined in a particular industry or for a specific population and the multiple adverse effects of having multiple definitions of *rural*.

**Conclusion:** The discrepancies found in current classification systems reveal the need for a standardized definition of *rural*. Ultimately, policies centered on securing health care services for rural populations are impacted by whatever definition of *rural* is used. Failing to establish a gold standard definition of *rural* could have harmful consequences to the health and wellbeing of the many people living in rural communities across the U.S.

## Introduction

The word *rural* conjures up an amalgam of images. Many liken *rural* to socially isolated and sparsely populated areas, with farmland that stretches as far as the eye can see, long dirt roads, and having a distinctive socio-cultural context with unique demographic configurations. While people claim to spot *rural* when they see it, most cannot define it and often overlook its complexity.^1^ Moreover, while it seems much more natural to depict what *rural* looks like in ordinary life, arriving at a precise and meaningful definition is much more challenging.^2,3^ Krout maintains that the term *rural* is often used so carelessly that it is virtually impossible to conclude its meaning.^4^ As a result, there is no standard or universal definition of *rural*,^5^ which partially explains why “at least 75 definitions of *rural*” (p. 5) are used by the U.S. government.^6^

Lacking a consensus definition at the federal level can affect rural communities in several ways. First, different sets of criteria can change the designation of any given area from rural to urban (or vice versa).^7^ Such interchangeable criteria can impact decisions about the number of resources available to communities, which may create and/or further health, education, and infrastructure disparities. Second, classifications of *rural* used by the federal government justify the planning, application, and monitoring of policy interventions and developmental efforts used to meet the needs of a population.^7,8^ This presents an exacerbation of resource issues when different federal agencies fund communities with department-specific definitions of *rural*. Third, lax standards for how areas are classified can make it difficult to justify and set aside resources to aid rural communities across the U.S.

Definitions of *rural* can also have significant impacts on health promotion and disease prevention. Rural classifications delineate how government functions in terms of policymaking, monitoring, and administration. In fact, definitions shape public policies, which create legislative action that can lessen impediments, build prospects, or offer motivations that influence health decisions.^9^ To that end, in 1987, Congress established the Federal Office of Rural Health Policy (FORHP) as part of the Health Resources and Services Administration (HRSA).^10^ FORHP oversees funding for rural health research, and it gives direct support to rural communities and state offices of rural health. Regardless of the overseeing agency, to qualify for rural-based initiatives aimed to improve population health, a county must be designated rural. Unfortunately, this can disqualify some counties and entities depending on what definition of *rural*is used.

For instance, a Critical Access Hospital in Northeast Georgia (GA) located in a county designated 99.25% rural might not qualify for the “Small Rural Hospital Improvement Program” (SHIP). They may not be eligible because HRSA, the federal overseer of SHIP, determines rural eligibility for this program based on location outside of a Metropolitan Statistical Area or within a rural census tract based on Rural-Urban Commuting Area (RUCA) code.^11^ Yet using these criteria, HRSA would designate the particular GA county as metropolitan. As a result, residents of this county could miss out on improved health practices, like telemedicine, that are supported through this program. In another example, Bennet and colleagues^10^ argue that since Metropolitan and Micropolitan Statistical Areas include urban and rural territories and populations, health research that uses urban-rural indicators, such as the Behavioral Risk Factor Surveillance System Survey, “leads to a large body of literature that depends upon an arguably poor measure of rurality” (p. 1986). These examples underscore the importance and the impact a definition of *rural* can have on public health efforts to prevent disease and illness.

Last, the multiple meanings of *rural* and lack of conceptual clarity make it challenging to have confidence in the conclusions formed by researchers.^12^ Hart and colleagues^5^ reported that “The use of noncongruent definitions of rural may result in markedly different conclusions and policy implications” (p. 1150), while Isserman^12^ stressed further that “When we get rural wrong, we reach incorrect research conclusions and fail to reach the people, places, and businesses our governmental programs are meant to serve” (p. 467). These are important considerations given that erroneous designations can result in inaccurate conclusions and policy recommendations that can negatively impact the nearly 20% of the total U.S. population that reside in rural areas.^13^ Thus, the lack of a uniform definition of *rural* can adversely affect rural communities by distorting the circumstances of what transpires within these communities as the definitions utilized are not necessarily appropriate for the research carried out. For example, failing to receive funds due to discrepancies in definitions of *rural* could unjustly affect health promotion efforts, health care services, poverty levels, and affordable housing options in rural communities.^14-17^

Only by appropriately defining *rural* can differences in health outcomes be determined with rural areas and between rural and urban areas.^5^ A clear understanding of how operational definitions of *rural* vary would assist policymakers, researchers, and rural practitioners to more appropriately identify and devise strategies that better meet the health needs of rural populations. Without a cohesive schema for *rural*, there is an inconsistency in how these communities are served. The purpose of this scoping review was to (1) determine how *rural* is being defined by the federal U.S. government and (2) establish a more nuanced understanding of what criterion has been used to designate an area rural. Despite the acknowledgment by policymakers and researchers that there are many definitions of *rural*, no extensive review of these various terms exists, to this research team’s knowledge.

## Materials and Methods

### Study design

A scoping review was selected over a systematic review because it addresses a more comprehensive range of topics specific to the subject matter and examines an array of varied study designs.^18^ Since one of the reasons for conducting scoping reviews is to “clarify concepts” (p. 1),^19^ the authors decided that this type of literature review would be more appropriate when examining the multiple definitions of *rural* used by the federal government. Additionally, by design, the focus of a scoping review was less narrow than a systematic review of the literature. Instead of reviewing a selection of articles with a specific focus, the review aims to “map rapidly the key concepts underpinning a research area and the main sources and types of evidence available and can be undertaken as [a] stand-alone [project]… especially where an area is complex or has not been reviewed comprehensively before” (p. 194).^20^ Consistent with other scoping reviews, the present study does not assess the quality of earlier studies, nor does it present a quantitative synthesis of data. Instead, it explored, captured, and clarified key definitions of *rural* found in the broader body of literature.

This scoping review was guided by Arksey and O’Malley’s 5-step process for synthesizing, analyzing, and summarizing relevant literature.^18^ In their seminal article, Arksey and O’Malley^18^ offered that “A key strength of the scoping study is that it can provide a rigorous and transparent method for mapping areas of research” (p. 30), and “present[s] the results in an accessible and summarized format, [so] policymakers, practitioners and consumers are better placed to make effective use of the findings” (p. 30). Because the discussion surrounding exactly how *rural* is defined has generated interest in not only articles but also conference presentations, the definitions of *rural* retrieved from this scoping review can be used as a reference for researchers and policymakers alike.

### Data analysis strategy

The first step in Arksey and O’Malley’s approach to conducting a scoping review is to *identify the research question*, which was, “What is known from the existing literature about how the U.S. federal government defines the term *rural*?”^18^ The second step in the process was *identifying relevant studies*. Since *rurality* can encompass many different attributes, a broad parameter was set to capture several different definitions in a wide array of studies. Several different online databases were used to search for a variety of documents. The authors consulted with the reference librarian at the authors’ home institution to determine which databases and journals would allow the most comprehensive scope of the multiple definitions of *rural*. It was decided that the university’s multi-search database would be used to gain access to over a hundred databases focusing on an assortment of subjects. Documents were selected from the online databases by searching for articles and reports that included some variation of the keywords *rural* and *define* in the title (for example, *rurality*and *definition* as well as *conceptualize*). After consulting with the reference librarian, the authors believed that these search terms would generate the most relevant documents for this study as they would be more likely to focus on ruraldefinitions.

Once an initial selection of documents was made, extraneous studies were filtered to fulfill the third step: *select studies*. This process was achieved by setting inclusion and exclusion published between the years 2000 and 2019. One of the primary definitions of *rural* is provided by the U.S. Census Bureau (USCB) which updates the definition decennially.^12^ Therefore, articles over 20 years old may be less relevant to existing definitions of *rural* and, consequently, current research surrounding how the term *rural* is defined. The second selection criterion established was that documents had to have been either published in the U.S. or, if published internationally, had to focus on how the U.S. government defined *rural*. Also, documents had to be printed in English. Finally, the documents had to emphasize the use of rural definitions, either by offering definitions of the term or stressing the differences between ruraltaxonomies used in research studies.

*Charting the data* is the fourth step in Arksey and O’Malley’s framework.^18^ Key themes were identified using a constant comparative method. To accomplish the task of *charting the data*, an Excel file was used to organize the themes that arose from the literature. Themes found were:

1. How *rural*is defined across a spectrum of fields
2. Positive and negative implications of having multiple definitions of *rural*
3. Reasons for why a conceptual definition of *rural* is needed
4. How a standardized definition of *rural*can be construed

The final step in this five-step process was *collating, summarizing, and reporting the results.*Arksey and O’Malley^18^ recommend that findings should be presented in two ways: through “the basic numerical analysis of the extent, nature and distribution of the studies included in the review” (p. 27), as well as thematically, “prioritiz[ing] certain aspects of the literature” (p. 27).

### Trustworthiness of data

Analyst triangulation was used to establish validity and corroborate the findings. The lead author and research assistant duplicated the third step, *study selection.* The lead author trained the research assistant to conduct the scoping review by developing a flowchart with detailed instructions. Each step was outlined with bullet points and emphasized searching for federal definitions of *rural* within the literature. After the data collection methods were duplicated, discrepancies were discussed between the two researchers. In all, only one federal definition was missed by the lead researcher, and after a thorough discussion, it was decided that this new definition would be included.

## Results

### Descriptive numerical summary

An initial search of the literature from the bibliographic databases produced 929 results using the search terms *rural* and some variation of the words *definition*(such as *define* and *defining)* or *conceptualize* in the title. This decision was made because documents with the words *define* or* conceptualize* and *rural* in their title would explicitly discuss the issues surrounding the matter, rather than just happen to have the terms *rural* and *define* in their abstract. Further evaluation of the retrieved documents revealed that after accounting for the filters (published between 2000 and 2019, U.S.-focused, and printed in English) and whether the definition of *rural* was emphasized, 49 documents met all criteria and were included in this scoping review. This final step eliminated articles that just happened to have *rural* and *define* in the title and did not necessarily address the definition of *rural*.

Overall, the 49 documents came from the following online library databases: Academic OneFile (14), InfoTrac (8), General OneFile (6), MedLine (3), Education Resources Information Center (2), Science Direct (2), Complimentary Index (2), Supplemental Index (2), British Library Document Supply Centre Inside Serials and Conference Proceedings (2), Global Issues in Context (1), Avery Index to Architectural Periodicals (1), Science Citation Index (1), EconLit (1), Academic Search Complete (1), Business Source Complete (1), Education Research Complete (1), and Library, Information Science & Technology Abstracts (1).

Of these 49 documents, including journal articles, newspaper articles, magazine articles, and reports, 42 were published in the U.S., and seven were published internationally. The selection resulted in journal articles, data files, and technical reports and spanned between 2000 and 2019. The publisher domains were delineated into the fields of health (10), education (8), geography (3), rural life (2), accident prevention (1), housing (1), business (1), congressional research (1), grassroots (1), and librarianship (1). The remaining 20 documents were news articles with topics specific to the implications of the definition of *rural*. Ultimately, 33 definitions of *rural* were gathered from the literature (See Tables 1, 2 and 3).

**Table 1:** Federal definitions of rural by population

**Population Based**	
**Defining body**	**Definition of rural**
Center for the Study of Rural Librarianship	“Defines a town of 25,000 individuals or less as rural” (p. 2).^21^
Community Development Block Grant Programs	“Define[s] rural as 50,000 or fewer people” (p. 1).^21^
Community Facilities Programs	“Territory outside Census places of 20,000 or more” (para. 7).^2^
Department of Education	“Any place determined by a state government to be rural”(para. 11).^22^
Farm Security and Rural Investment Act of 2002	**General Definition**- “The terms “rural” and “rural area” mean any area other than (i) a city or town that has a population of greater than 50,000 inhabitants; and (ii) the urbanized area contiguous and adjacent to such a city or town”(p. 496).^12^
	**Water and Waste Disposal Grants**- “The terms “rural” and “rural area” mean a city, town, or unincorporated area that has a population of no more than 10,000 inhabitants” (p. 496).^12^
	**Rural Business Investment**- “(i) Outside a standard metropolitan statistical area; or (ii) within a community that has a population of 50,000 inhabitants or less” (p. 496).^12^
	**National Rural Development Partnership**- “(i) All the territory of a state that is not within the boundary of any standard metropolitan statistical area” and “(ii) all territory within any standard metropolitan statistical area within a census tract having a population density of less than 20 persons per square mile” (p. 496).^12^
Frontier and Remote Area codes	“FAR areas are defined in relation to the time it takes to travel by car to nearby Urban Areas (UAs), defined by the Census Bureau to identify densely settled urban territory in a nationally consistent manner… Level 1—FAR areas consist of rural areas and urban areas up to 50,000 people that are 60 minutes or more from an urban area of 50,000 or more people. Level 2—FAR areas consist of rural areas and urban areas up to 25,000 people that are: 45 minutes or more from an urban area of 25,000-49,999 people; and 60 minutes or more from an urban area of 50,000 or more people. Level 3—FAR areas consist of rural areas and urban areas up to 10,000 people that are: 30 minutes or more from an urban area of 10,000-24,999; 45 minutes or more from an urban area of 25,000-49,999 people; and 60 minutes or more from an urban area of 50,000 or more people. Level 4—FAR areas consist of rural areas that are: 15 minutes or more from an urban area of 2,500-9,999 people; 30 minutes or more from an urban area of 10,000-24,999 people; 45 minutes or more from an urban area of 25,000-49,999 people; and 60 minutes or more from an urban area of 50,000 or more people” (para. 2).^2^
Library Services and Construction Act	“Communities with populations of 10,000 or less” (p. 2).^21^
National Center for Education Statistics	**Metro Centric Locale Codes**- “Two rural codes (outside MSA, inside MSA) based on proximity to MSA. Locations must be census-defined rural territory” (p. 7).^23^
	**Urban Centric Locale Codes**- “Three rural codes (fringe, distant, remote) based on distance from urbanized area. Rural schools and districts must be census-defined rural territory” (p. 7).^23^
Office of Management and Budget	“Rural areas are non-core areas (i.e., regions outside metropolitan and micropolitan statistical areas). Metropolitan statistical areas include counties or county clusters containing at least one urban area with a population of at least 50,000. Micropolitan statistical areas contain an urban area of 10,000-49,000 people” (p. 179).^24^
Rural Development Single-family and Multifamily Housing Loan and Grant Program	“Has a population that does not exceed 35,000 and is rural in character”(para. 3).^25^
Rural Electrification Act	“Considers communities of 1,500 or fewer people to be rural” (p. 1).^21^
Rural-Urban Commuting Area (RUCA) codes	“Large Rural Towns have Micropolitan cores and substantial commuting patterns to Urban Clusters. Small Rural Towns have primary commuting flows to or within population centers of between 2,500 and 9,999 residents. Isolated Rural Towns are less populated rural areas with no primary commuting flows to Urbanized Areas or Urban Clusters”(p. 302).^26^
Rural Urban Continuum Codes (RUCCs)	“The non-metropolitan areas are subdivided into six groups (Codes 4-9) based on their population size and proximity to a metropolitan area” (p. 180).^24^
Social Security Act	“A “large urban area” means… an urban area which… has a population of more than 1,000,000… and the term “rural area” means any area outside such an area or similar area” (p. 496).^12^
United States Census Bureau	“The Census Bureau partitions urban areas into urbanized areas and urban clusters. The same census tract-based criteria are used for both; however, the urbanized areas have cores with populations of 50,000 or more, and the urban clusters have cores with populations that range from 2,500 to 49,999. All other areas are designated as rural” (p. 1151).^5^
U.S. Department of Agriculture, Forest Service	“Rural communities… with a total population of 10,000 or less... county, special district, and/or other local unit of government, that is not contained within a Metropolitan Statistical Area” (p. 496).^12^
U.S. Department of Agriculture, Economic Research Service	**Urban Influence Codes**- “[N]on-metropolitan counties are grouped according to their adjacency and nonadjacency to metropolitan counties and the size of the largest urban settlement within the county” (p. 1151).^5^
U.S. Department of Agriculture, Rural Development	**General Definition**- “[Areas] eligible for Rural Development Funding… as long as that community me[ets] population requirements of below 25,000” (para. 3).^27^
	**Rural Housing Repair and Rehabilitation**- “Home must be located in a non-metropolitan area. Non-metropolitan area includes rural areas and communities of less than 20,000 persons” (p. 496).^12^
	**Rural Broadband Access Loan-** “Community being served has a population less than 4,000” and population density is “not more than 20 persons per square mile” (p. 496).^12^
	**Rural Business Enterprise Grants**- “Any area other than a city or town that has a population of greater than 50,000 inhabitants and the urbanized area contiguous and adjacent to such a city or town” (p. 496).^12^
	**Distance Learning and Telemedicine**- “Exceptionally Rural Area, area not within a city, village or borough, ≤ 5000 population... Rural Area, incorporated or unincorporated area, > 5000 and ≤ 10,000 population... Mid-Rural Area, incorporated or unincorporated area, > 10,000 and ≤ 20,000 population”(p. 496).^12^
U.S. Department of Health and Human Services	“All counties that are not designated as parts of Metropolitan Areas are considered rural” and “Census tracts with RUCA codes 4 through 10 are considered rural” (p. 496).^12^
U.S. Department of Housing and Urban Development	“A place having fewer than 2,500 inhabitants, a county with an urban population of 20,000 inhabitants or less, territory, persons and housing units in the rural portion of “extended cities,” open country that is not a part of or associated with an urban area, any place with a population not in excess of 20,000 inhabitants and not located in a Metropolitan Statistical Area” (p. 496).^12^
U.S. Department of Transportation, Federal Transit Administration	“Non-urbanized areas, < 50,000 in population” (p. 496).^12^
Veteran’s Health Administration	“Census tracts that belong to Urbanized Areas are designated as Urban locations; all other locations are considered Rural, except for those in counties with average population density of less than 7 residents per square mile, which are designated as Highly Rural” (p. 302).^26^

**Table 2:** Federal definitions of rural by population density

**Population Density Based**	
**Defining body**	**Definition of rural**
Department of Education	“Any place determined by a state government to be rural” (para. 11).^22^
Farm Security and Rural Investment Act of 2002	**National Rural Development Partnership**- “All the territory of a state that is not within the boundary of any standard metropolitan statistical area” and “all territory within any standard metropolitan statistical area within a census tract having a population density of less than 20 persons per square mile” (p. 496).^12^
Flash Flood Monitoring and Prediction	“Reports “rural residential land” at densities of 2 to 0.5 units per acre” (p. 58).^28^
Frontier and Remote Area codes	“FAR classiﬁes Census tracts (or zip codes, optionally) based on their population density and the travel time required to reach urban areas… Level 1—FAR areas consist of rural areas and urban areas up to 50,000 people that are 60 minutes or more from an urban area of 50,000 or more people. Level 2—FAR areas consist of rural areas and urban areas up to 25,000 people that are: 45 minutes or more from an urban area of 25,000-49,999 people; and 60 minutes or more from an urban area of 50,000 or more people. Level 3—FAR areas consist of rural areas and urban areas up to 10,000 people that are: 30 minutes or more from an urban area of 10,000-24,999; 45 minutes or more from an urban area of 25,000-49,999 people; and 60 minutes or more from an urban area of 50,000 or more people. Level 4—FAR areas consist of rural areas that are: 15 minutes or more from an urban area of 2,500-9,999 people; 30 minutes or more from an urban area of 10,000-24,999 people; 45 minutes or more from an urban area of 25,000-49,999 people; and 60 minutes or more from an urban area of 50,000 or more people” (para. 2).^2^
Health Resources and Services Administration	**Federal Office of Rural Health Policy**- “Accepts all non-metro counties as rural and uses an additional method of determining rurality called the Rural-Urban Commuting Area (RUCA) codes… These are based on Census data which is used to assign a code to each Census Tract. Tracts inside Metropolitan counties with the codes 4-10 are considered rural” (para. 14).^22^
Rural-Urban Commuting Area (RUCA) codes	“Large Rural Towns have Micropolitan cores and substantial commuting patterns to Urban Clusters. Small Rural Towns have primary commuting flows to or within population centers of between 2,500 and 9,999 residents. Isolated Rural Towns are less populated rural areas with no primary commuting flows to Urbanized Areas or Urban Clusters” (p. 302).^26^
United States Census Bureau	“The Census Bureau partitions urban areas into urbanized areas and urban clusters. The same census tract-based criteria are used for both; however, the urbanized areas have cores with populations of 50,000 or more, and the urban clusters have cores with populations that range from 2,500 to 49,999. All other areas are designated as rural” (p. 1151).^5^
U.S. Department of Agriculture, Rural Development	**Rural Broadband Access Loan**- “Community being served has a population less than 4,000” and population density is “not more than 20 persons per square mile” (p. 496).^12^
Veteran’s Health Administration	“Census tracts that belong to Urbanized Areas are designated as Urban locations; all other locations are considered Rural, except for those in counties with average population density of less than 7 residents per square mile, which are designated as Highly Rural” (p. 302).^26^

**Table 3:** Federal definitions of rural by urban integration

**Urban Integration**	
**Defining body**	**Definition of rural**
Department of Education	“Any place determined by a state government to be rural” (para. 11).^22^
Farm Security and Rural Investment Act of 2002	**General Definition**- “The terms “rural” and “rural area” mean any area other than (i) a city or town that has a population of greater than 50,000 inhabitants; and (ii) the urbanized area contiguous and adjacent to such a city or town” (p. 496).^12^
Frontier and Remote Area codes	“FAR classiﬁes Census tracts (or zip codes, optionally) based on their population density and the travel time required to reach urban areas… Level 1—FAR areas consist of rural areas and urban areas up to 50,000 people that are 60 minutes or more from an urban area of 50,000 or more people. Level 2—FAR areas consist of rural areas and urban areas up to 25,000 people that are: 45 minutes or more from an urban area of 25,000-49,999 people; and 60 minutes or more from an urban area of 50,000 or more people. Level 3—FAR areas consist of rural areas and urban areas up to 10,000 people that are: 30 minutes or more from an urban area of 10,000-24,999; 45 minutes or more from an urban area of 25,000-49,999 people; and 60 minutes or more from an urban area of 50,000 or more people. Level 4—FAR areas consist of rural areas that are: 15 minutes or more from an urban area of 2,500-9,999 people; 30 minutes or more from an urban area of 10,000-24,999 people; 45 minutes or more from an urban area of 25,000-49,999 people; and 60 minutes or more from an urban area of 50,000 or more people”(para. 2).^2^
National Center for Education Statistics	**Metro Centric Locale Codes**- “Two rural codes (outside MSA, inside MSA) based on proximity to MSA. Locations must be census-defined rural territory” (p. 7).^23^
	**Urban Centric Locale Codes**- “Three rural codes (fringe, distant, remote) based on distance from urbanized area. Rural schools and districts must be census-defined rural territory” (p. 7).^23^
Rural-Urban Commuting Area (RUCA) codes	“Large Rural Towns have Micropolitan cores and substantial commuting patterns to Urban Clusters. Small Rural Towns have primary commuting flows to or within population centers of between 2,500 and 9,999 residents. Isolated Rural Towns are less populated rural areas with no primary commuting flows to Urbanized Areas or Urban Clusters” (p. 302).^26^
Rural Urban Continuum Codes (RUCCs)	“The non-metropolitan areas are subdivided into six groups (Codes 4-9) based on their population size and proximity to a metropolitan area” (p. 180).^24^
U.S. Department of Health and Human Services	“All counties that are not designated as parts of Metropolitan Areas are considered rural” and “Census tracts with RUCA codes 4 through 10 are considered rural” (p. 496).^12^
U.S. Department of Agriculture, Economic Research Service	**Urban Influence Codes**- “[N]on-metropolitan counties are grouped according to their adjacency and nonadjacency to metropolitan counties and the size of the largest urban settlement within the county”(p.1151).^5^

### Qualitative thematic analysis results

#### Organizational focus

A more rigorous analysis revealed that the literature could be separated into two categories. The first category focuses on how *rural* was defined in a particular industry or for a specific population (such as nursing and epidemiology studies or for veterans and motorists). The second category is the clear implications of a particular definition or multiple definitions of *rural*; multiple inferences were likely to be depicted negatively, with many documents emphasizing the adverse effects of having multiple definitions of *rural*. These articles demonstrate how multiple definitions impact research on particular subjects, such as those mentioned above. The articles would often utilize multiple definitions of the term to compare statistical results and determine whether there were any inconsistencies in the findings, for example, as with the article “Defining the Rural HIV Epidemic: Correlations of 3 Definitions—South Carolina, 2005-2011,” by Weissman et al.^29^ This comparison process was also used to examine discrepancies in rate differences in special education practices, home health care usage, and veteran health care planning, depending on which definition of *rural* was utilized.^23,24,26,29^

### Conceptualization of ‘rural’

There were three main ways the federal government determined whether an area was considered rural: population size, population density, and integration to an urban area (see Tables 1, 2, and 3). Overall, of the 33 definitions extracted, 30 included population thresholds, ten included population density requirements, and nine were somehow integrated with urban areas, for instance, urban adjacency conditions. As was the case of Frontier and Remote Area (FAR) codes, sometimes a definition included all three stipulations. FAR codes dictate that for an area to be considered *urban* or *rural* depends on population, population density, and the travel time needed to reach an urban area.^2^ Additionally, classification schemes were distinguished at the county level or the sub-county level. An example of a county-level rural schema is the Office of Management and Budget’s (OMB) distinction of metropolitan and micropolitan.^30^ In contrast, an example of a sub-county level classification is the USCB definition based on an area’s population at the census tract or block level.^23^

### Federal definitions of ‘rural’

Results of the scoping review reflect 33 definitions of *rural* used by the federal government. The definitions of *rural* span the following 9 different federal agencies: the USCB, U.S. Department of Agriculture, U.S. Department of Education, U.S. Department of Health and Human Services, U.S. Department of Housing and Urban Development, U.S. Department of Transportation, Veteran’s Health Administration, National Rural Development Partnership, and OMB. Acts of legislation also included definitions of *rural* and spanned the Library Services and Construction Act, Consolidated Appropriations Act, Farm Security and Rural Investment Act, Social Security Act, and the Rural Electrification Act. A review of the literature revealed that the most common definitions of *rural* were those provided by the USCB and OMB, a claim also substantiated by HRSA.^24^

The findings reveal that on multiple occasions, the same agency reported several different departmental definitions of *rural*. For instance, the USDA alone offered 11 definitions of *rural*, dispersed across various departments. Similarly, the Farm Security & Rural Investment Act also included four definitions of *rural*. The remaining definitions found in this scoping review were disseminated across programs, centers, and codes, including the Center for the Study of Rural Librarianship, Department of Education National Center for Health Statistics, National Center for Education Statistics, Community Facilities Programs, Community Development Block Grant Programs, and FAR codes. Of the 33 definitions extracted from the literature, 30 of them included a population limit stipulation. As evidenced by the number of federal definitions that require an area’s population to be under a certain population threshold, clearly the U.S. federal government equates *rural* to population; however, the specific population limit was not uniformly defined.

Of the 30 definitions that included population parameters, there were nine different population limits: 1500, 2500, 4000, 10 000, 20 000, 25 000, 35 000, 50 000, and 1 000 000. Of these limits, the populations were divided as follows: 1500 (3%), 2500 (3%), 4000 (3%), 10 000 (13%), 20 000 (19%), 25 000 (7%), 35 000 (3%), 50 000 (45%), and 1 000 000 (3%). Further complicating matters is the variability in what constitutes an “area” for rural definitions using a population threshold. For example, in some instances, an area was distinguished by either county, territory, geographical area, community, place, town, city, open country, or village. Subtle nuances such as these make it even more difficult for areas to obtain resources that might be otherwise available to them. Such differences in population stipulations make it challenging for rural areas to receive adequate health resources to combat public health emergencies, such as what is being experienced in the U.S.’ handling of the coronavirus (COVID-19) pandemic.

### U.S. Census Bureau 

The scoping review revealed that the definition put forth by the USCB was one of the most used rural taxonomies in epidemiology, education, and health policy and was used multiple times in the literature when contrasting rural definitions.^12,13,31^ However, the USCB’s definition of *rural*was appropriated by the absence of urban.^31^ In other words, that which is not *urban* is *rural*. The USCB identifies *urban* using a sophisticated algorithm, considering the population of an area and density.^31^ Ultimately, a census block is separated into either an urban area (UA) or an urban cluster (UC). UAs have populations over 50 000 people, while a UChas a population between 2500 and 49 999.^5^ Per Hall and colleagues,^31^ the 2000 USCB defines *urban* as either a UA or a UC and “consist[s] of core census block groups or blocks that have a population density of at least 1000 persons per square mile AND surrounding census blocks that have an overall density of at least 500 people per square mile” (p. 165). Any census block not designated as a UA or a UC was, by default, considered ruralby the USCB.^31^

UCs are relatively new to the USCB’s designation of rural, only introduced in the 2000 census.^31^ While the inclusion of the UC identifier allows for a more granular assessment of an area, longitudinal assessments of census data are not easily comparable as the introduction makes for inconsistent comparisons across time periods. Modifications to the USCB’s definition were made on a decennial basis, further complicating the ability to assess data over a period of time and to make comparisons between rural studies. Modifications were also made to definitions put forth by the OMB, USDA, National Center for Education Statistics, Rural Urban Commuting Codes, and Veteran’s Health Administration. By contrast, some definitions of *rural*, like the one stated in the Library Services and Construction Act, have not been updated in nearly 60 years.^21^

Furthermore, because the USCB’s definition was contingent upon population density, UAs may extend across multiple counties and states.^24^ Despite these issues, there were strong points in the USCB’s definition, mainly the extent to which the definition has been used. The vast degree to which the USCB definition has been utilized in research permits data using this classification system to be compared between studies.^24^ Additionally, this taxonomy allows researchers to conduct studies that span geographical regions and provide the ability to aggregate data at the county level.^24,26^

### Office of Management and Budget

The OMB offered the most utilized county-level schema and was often applied as the foundation for more nuanced definitions that establish eligibility and reimbursement rates for over 30 federal programs.^5,26^ This scheme informed federal and state policies with large-scale implications for areas identified as *rural* by the OMB.^24^ While updated less regularly or consistently, the OMB classiﬁcation system was most recently changed in 2003, modifying the existing deﬁnition of *urban* from Metropolitan Statistical Areas (MSAs) to Core Based Statistical Areas (CBSAs). CBSAs were divided into either MSAs or Micropolitan Statistical Areas. MSAs include counties or county clusters with “at least one urban area with a population of at least 50 000 [and]… micropolitan statistical areas containing an urban area of 10 000-49 000 people. Rural areas are non-core areas (i.e., regions outside metropolitan and micropolitan statistical areas)” (p. 179).^24^ The OMB^32^ suggested that they define metropolitan and micropolitan statistical areas “to provide nationally consistent definitions for collecting, tabulating, and publishing federal statistics” (para. 2).

Strengths of OMB’s delineation include consistent geographical boundaries since counties are not changed often and the ability to make finer distinctions between areas.^24^ It has been maintained that weaknesses to the OMB model were mainly due to using counties as the geographical unit for determining an area as *urban* or *rural*.^24^ Because of the population density variability in large counties, there was a risk for rural areas to be absorbed by MSAs, potentially limiting the availability of resources and funding. Given that rural areas may be concealed within larger metropolitan counties, research specific to rural populations can be difficult. Additionally, it is challenging to establish variance between groups by including multiple geographical areas within one unit of analysis.

Another weakness of the definition offered by the OMB was that people tend to consider the metropolitan and micropolitan areas that make CBSAs *urban* and those non-core areas as *rural*. Using *urban* and *rural* interchangeably can be misleading, as micropolitan areas, or counties with populations between 10 000-50 000, can be more representative of rural areas than the urban areas they are more frequently associated.^24^ By using these terms interchangeably, we ultimately fall into what Isserman refers to as a “county trap,” where all metropolitan counties are considered urban areas, and all other areas are considered rural.^12^ Falling into this trap, the overlap of urban and rural populations is disregarded.^12^ While the USCB and OMB remain the leading authorities of defining *urban*and *rural*, the imprecision of the methods used to distinguish these classifications suggests that an improved and more comprehensive approach is needed to conduct and implement useful research and policy.

## Discussion

There are significant challenges facing rural communities and the open dialogue surrounding understanding just what is *rural*, in what context, and by whose standards. The present scoping review produced 33 different federal ruraldefinitions. Although the literature has shown some tables that reflect the multiple definitions of *rural*, the present analysis revealed that the largest table was in Isserman’s article, which revealed 15 different definitions of *rural*; however, that article was published over 15 years ago.^12^ The results of both studies represent a guide that researchers can use when geographically mapping the populations they want to study and for what purpose.

This study revealed that definitions of *rural* tend to vary across agencies and ultimately across geographical locations; therefore, it is critical to ensure accuracy in the populations being measured. Hawley and colleagues^23^ noted that “by explicitly defining rural, researchers better operationalize their construct, hereby enabling future researchers to evaluate and critique the alignment between different conceptualizations of rural” (p. 9). After the literature was canvassed, the findings suggest that rural definitions are defined by population, population density, or their integration to urban areas, but questions remain as to whether these available definitions truly capture what defines rurality. For example, how does the diversity of definitions impact rural populations? Future research should explore the kind of impact experienced by rural populations when definitions change, as they often do.

This review suggests that the federal government has largely ignored culture, values, and norms as indicators of rural areas; however, *rural* encompasses culture in addition to demography and geography.^33^ While most federal deﬁnitions construe rurality as a location rather than a lifestyle, a more comprehensive approach for identifying *rural* allows for “an understanding of the complexities of the culture, way of life, and state of mind associated with rurality” (p. 176).^24^ This is important because the cultural context is a prominent force that molds attitudes and tenets.^34^ Culture presents a milieu through which worldviews are constructed and shapes how people make sense of the world in which they live and the relationships in which they engage.^35,36^ Thus, it is suggested that researchers and federal government offices should consider culture as a component of their study when defining a rural population or area. Failing to recognize its importance can ultimately influence findings and policy, adversely impacting the rural individuals’ cultures living in these areas.^24^

Knowing the available definitions of *rural*can contribute to a better understanding of how rural populations can be aided. Hawley and colleagues^23^ reported that rural definitions should “be compared and contrasted in order to provide guidance as to which definition may be most appropriate for a given context and to help research consumers synthesize findings across studies that use different rural definitions” (p. 3). This scoping review provides guidance as to which definition may be most appropriate for a given context by exposing the public to how many definitions of *rural* exist—even within the same agency and the characteristics that make up these taxonomies. To this research team’s knowledge, no other reviews examine how the U.S. government defines *rural* as extensively as this one. When considering which definition of *rural* will be used in the emerging stages of the research process, the definitions outlined in this scoping review can be used as a starting point for this critical step.

### Implications of multiple rural definitions

The multiple definitions and measures of *rural* complicate how rural areas are characterized, and thus, how resources are allocated. This is especially apparent when looking at ruraldefinitions whose contributing factor is population threshold, the most common way *rural* is depicted by the U.S. government. However, the difference between a population range of 1500 and 1 000 000 people is concerning because areas considered rural by one definition might be considered urban by another, making some areas ineligible for federal resources. The inconsistencies in taxonomies could contribute to rural areas missing out on programs and resources that may be vital to their success and the health of the people who live there. For example, Hart and colleagues^5^ maintain that “A town with a population of 3000 in a very remote area is considered urban under the Census Bureau definition, but that same town is often nonmetropolitan under the OMB definition” (p. 1153). As a result, areas can be under-classified as rural. These classification inconsistencies can have adverse consequences for health promotion and disease prevention efforts.

The COVID-19 pandemic is a crucial example: screening and testing for COVID-19 remain not readily available across rural areas in the U.S. However, as the pandemic has reached rural areas, it has overwhelmed the smaller health care structures, making treatment of this virus difficult to manage. Some of these situations could have been circumvented if not for a discrepancy in how these areas were categorized. Having so many rural meanings can complicate matters because some definitions are updated on a decennial or otherwise on a random basis. As a result, researchers who use a definitive description of *rural* research may find that the definition has been updated, adversely affecting how data is measured and interpreted over time. This can be especially concerning when looking at health outcomes, population health data, and public health program research since data cannot be compared longitudinally.

### Implications for rural health research

Distinguishing the characteristics of rural and urban populations is essential because the differentiation provides researchers with a better understanding of the nuances specific to rural communities.^12,23^ Definitions that do not differentiate between levels of rurality may obscure emerging problems at the local level by aggregating rural areas of differing sizes and levels of remoteness. Consequently, public policies may fail to appropriately target intra-rural populations.^5^ Hart and colleagues^5^ maintain further that, “By treating these diverse types of rural cities and towns and the problems they confront similarly, policy analysts may fail to identify each site’s distinct health care concerns and effective methods for resolving those problems” (p. 1149).

These inconsistencies translate into research when rural phenomena are explained, potentially causing these occurrences to be broader, narrower, or a completely different phenomenon based on the definition of *rural*used.^23^ The classification of *rural* used will influence policymakers decision to allocate resources and funding for health services, such as primary and emergency care, surgery, public health initiatives, and telemedicine expansion. As a result, funding could be concentrated in more populated areas.^26^ Thus, scientists and policymakers should be purposeful and discerning when delineating rural taxonomies associated with rural health promotion intervention, and research efforts.^24^

The differences in how areas are classified as rural is also problematic for rural health promotion and policy. The prevalence of certain health conditions, which could have implications for policy and program development to address the disparities experienced in these areas, can fluctuate depending on the definition of *rural* applied. These discrepancies have major implications when looking at the prevalence of disease as well as health outcomes. The prevalence and mitigation of disease can be impacted by policies and programs, but it is crucial that the intended population being studied is in fact represented by the data used. Bennet and colleagues^10^ present this occurrence using Maine and Indiana as an example.

Nearly the same geographical size, Maine has only 16 counties while Indiana has almost six times that many, with 92. Consequently, the urban counties in Maine are expansive enough to include rural areas, with some of these areas as far as 100 miles away from the urban center.^10^ This could mean that these individuals are also up to 100 miles away from major hospitals and specialty care. When Bennett and colleagues^10^ compared a county level definition with rural RUCA codes to examine pregnancy discharge data, there was an 18% difference in the number of rural deliveries. Similarly, a study that examined newly diagnosed HIV patients in rural South Carolina demonstrated new HIV cases varied from 23.3% to 32.0% depending on the rural definition used.^21^ The discrepancies in populations captured in both of these examples are large enough to potentially impact health policy.

Finally, the use of a particular rural definition not only impacts how health research around these populations occurs but the populations themselves can be affected. For example, the fundamental purpose of telemedicine is to increase access to health care services, ultimately with the goal of improving health outcomes. There are several programs that support telemedicine expansion in rural areas, for instance, the Rural Health Care Telecommunications Program. This program determines rural eligibility with a “Look Up Tool” that uses a county level definition. As explained before, there are limitations to this approach, and thus, some rural populations living in urban counties might miss out on valuable health promotion programs and services they otherwise might be eligible for had another definition of *rural* been used.

Because of the multiple definitions of *rural* across research studies, different interpretations of rural health data are engendered.^37^ Policymakers whose work surrounds securing health care services for rural populations will be impacted by whichever definition of* rural*is used.^26^ These discrepancies in the definition of *rural*, if any is used at all, challenge the comparison of findings across studies and influence data interpretation.^24^ The need to come to a standardized definition of *rural*is not only necessary when carrying out research that includes rural populations, but it is ethically responsible if this underserved population is indeed going to be adequately served.

### The argument for a standardized definition of ‘rural’

There are apparent drawbacks to a dichotomized understanding of *urban*and *rural*, which is the most utilized classification system for defining these areas from this study’s findings. By dichotomizing urban-rural classifications, many federal agencies and legislative codes conceal heterogeneity, “lack[ing] sensitivity to local variations in rural areas” (p. 521).^37^ Conventional methods used to distinguish a rural area are insufficient because the dichotomized definition makes it impossible to study a well-defined population as these methods do not account for the variation of population density within large areas. Furthermore, there are significant inconsistencies between the definitions presented by the USCB and OMB, two of the most widely accepted rural designations. Using different definitions to identify rural populations is problematic since each taxonomy was developed for different reasons and geographical areas. Dependent on the definition used, individuals who live in low population density areas may be classified urban by the USCB and rural by the OMB if they are adjacent or near an urban area.^24^ These discrepancies are one example of how a standardized definition of *urban* and *rural*would minimize the ambiguity across federal agencies.

It has been argued that the variations of rurality make it challenging to propose a standardized definition. The argument is not illogical since, depending on how the definition is structured, a standardized definition of *rural* may overlook some rural populations. This may be of particular concern today in the U.S. because of the shifting demography occurring in many rural communities.^38^ Researchers can help minimize this issue by adequately describing the socio-cultural aspects of a study’s examined population. This ensures that consumers of research are informed of the people and areas examined and allows for the correct interpretation of findings and accurate comparisons between studies. The benefits of a definition that can be used across multiple agencies and research studies outweigh the risks.

### Future considerations

Working toward a standardized definition of *rural* might help eliminate some of these discrepancies and confusion. Researchers have made a point that when the term *rural* is used, it is unclear *whose* definition of rural is applied. As evidenced by the 33 definitions of *rural* found in this scoping review, the many taxonomies make it difficult to impossible to know which definition was considered (or applied) in any given study or government document. By not understanding what *rural* means, we cannot truly understand the people that live there.

A standardized hierarchal definition that recognizes multiple populations and population density thresholds of what constitutes *rural* would minimize some of the issues associated with the present delineation of definitions, making the data of future longitudinal rural research studies more reliable. Furthermore, by offering a granular definition of *rural*, a multi-level analytical approach could be applied, possibly gaining a better understanding of the nuances reflected in various populations.^39^ Hart and colleagues^5^ suggest that “An appropriate rural and urban taxonomy should (1) measure something explicit and meaningful; (2) be replicable; (3) be derived from available, high-quality data; (4) be quantifiable and not subjective, and (5) have on-the-ground validity” (p. 1150).

As a component of this scoping review, a four-question survey was conducted during Summer 2019 that examined how *rural* is defined by researchers, educators, professionals of rural populations, and the general public. An optional sixth step in Arksey and O’Malley’s process of conducting a scoping review, *consulting the experts*, is carried out to glean further inside knowledge on a topic.^18^ By learning how the term *rural*is construed by these four subsets, this data can be aligned with the definitions and data culled from the current scoping review to present a conceptual definition of *rural* to be used across agencies for years to come. Inclusion of a public survey can help contribute to a better representation of the perception of what *rural* entails. After all, the former U.S. Secretary of the USDA himself, Sonny Perdue,^40^ stated when he testified to the Senate Appropriations Committee in 2019 that “We would love to have a comprehensive definition of rural” (p. 61). Appealing to the Committee, Perdue^40^ encouraged them to:

“Look at a common definition of rural that you could direct in many of our programs regarding access. We are limited to defining rural as under 20,000 in many places, under ten in some other places. We would love to have a common definition because the places that might have been 10,000 10 years ago may be 20,000 now, and those who might have been 20,000 are now 40,000 and 50,000 and still need help many times in their growth, water, water treatment plants and others in a more definitive way” (p. 61).

We suggest that responses gathered from ruralexperts and professionals who participate in the national survey will help develop a conceptual framework of what constitutes rurality and further explore the unbeaten path of establishing a standardized definition of *rural*.

A few limitations should be acknowledged. Although 33 definitions of *rural* were extracted during this scoping review, there may still be many more definitions used by government agencies and research teams. Other definitions of* rural* may exist but were not accounted for in this review because grey papers were excluded. If U.S. rural demographer Calvin Beale was accurate, the difference can be as vast as nearly 40 definitions, since he claimed at least 75 definitions of *rural*exist.^6^ Additionally, while there were considerable benefits of using a multi-search system, not *every* article written about rural taxonomies was pulled, and thus, it is likely that contributing documents with relevant information were missed. This is especially true since there may be articles that used a different variation of the term *definition* or *conceptualize*.

## Conclusion

Since the USCB first defined *urban* in 1910,^41^ the concept of *rural* has evolved as much as the landscape itself. Throughout the federal government, the myriad definitions of *rural* have led to issues arising from having varied meanings of the term, including the misclassification of rural areas as urban.^5^ The number of and discrepancies between rural definitions can significantly impact research, resource allocation, funding, and policy formation in rural communities.^23^ Ultimately, policies centered on securing health promotion and health care services for rural populations are impacted by whatever definition of *rural* is used.^26^ As a result, more research is needed to determine how a gold standard definition of *rural* can be established to fairly and better meet the needs of rural populations. Failing to do so could have harmful consequences to the health and wellbeing of the many people living in rural communities across the U.S.

## Authors’ contributions

EMC established the concept behind the scoping review, conducted the scoping review and analysis, initiated and edited the manuscript and prepared charts and references. JFB assisted with manuscript development and preparation, while JRB assisted with data review, classification assistance, and data analysis, which was instrumental in triangulating the results achieved. Each author has reviewed, approved, and consented to this manuscript’s submission, and claim accountability for its accuracy and integrity in relation to the criteria set forth by ICME.

## Funding

No external funding was obtained for this study.

## Ethical approval

This review does not require ethical approval since it was an analysis of peer-reviewed publications and did not involve human participants.

## Competing interests

The authors declare no conflicts of interest.
